# Using Diffusion-Weighted MRI to Predict Central Lymph Node Metastasis in Papillary Thyroid Carcinoma: A Feasibility Study

**DOI:** 10.3389/fendo.2020.00326

**Published:** 2020-06-12

**Authors:** Heng Zhang, Shudong Hu, Xian Wang, Wenhua Liu, Junlin He, Zongqiong Sun, Yuxi Ge, Weiqiang Dou

**Affiliations:** ^1^Department of Radiology, Affiliated Hospital, Jiangnan University, Wuxi, China; ^2^Department of Radiology, Affiliated Renmin Hospital, Jiangsu University, Zhenjiang, China; ^3^Department of Radiology, Tinglin Hospital of Jinshan District, Shanghai, China; ^4^GE Healthcare, MR Research China, Bejing, China

**Keywords:** thyroid cancer, diffusion magnetic resonance imaging, lymphatic metastasis, nomograms, feasibility studies

## Abstract

**Objective:** To investigate whether diffusion-weighted imaging (DWI) with multi b values can be used as a quantitative assessment tool to predict central lymph node metastasis (CLNM) in papillary thyroid carcinoma (PTC).

**Method:** A total of 214 PTC patients were enrolled from January 2015 to April 2018. Each patient underwent multi b value DWI (300, 500, and 800 s/mm^2^) preoperatively and then clinical treatment of central LN dissection at the Thyroid Surgery Department. These patients were divided as two groups based on with and without CLNM. The corresponding apparent diffusion coefficients (ADCs) were evaluated with separated b value, i.e., 300, 500, or 800 s/mm^2^. Clinicopathological variables and ADC values were analyzed retrospectively by using univariate and binary logistic regression. The corresponding obtained variables with statistical significance were further applied to create a nomogram in which the bootstrap resampling method was used for correction.

**Results:** PTCs with CLNM had significantly lower ADC_300_, ADC_500_, and ADC_800_ values compared with PTCs without CLNM. Using receiver operating characteristic (ROC) analysis, the ADC_500_ value (0.817) showed a higher area under the curve (AUC) than those of the ADC_300_ and ADC_800_ values (0.610 and 0.641, respectively) in differentiating patients with CLNM and without CLNM. The corresponding cutoff value for ADC_500_ was also determined (1.444 × 10^−3^ mm^2^/s), with respective sensitivity and specificity of 88.6 and 66%. The nomogram was generated by binary logistic regression results, incorporating four variables: gender, primary tumor size, extrathyroidal extension (ETE), and ADC_500_ value. The AUC of the nomogram was 0.894 in predicting CLNM. Moreover, as shown in the calibration curve between nomogram and clinical findings, a strong agreement was observed in the prediction of CLNM.

**Conclusions:** In summary, the ADC value is a valuable noninvasive imaging biomarker for evaluating CLNM in PTCs. The nomogram, as a clinical predictive model, is able to provide an effective evaluation of CLNM risk in PTC patients preoperatively.

## Introduction

Papillary thyroid carcinoma (PTC) as the most common type of thyroid malignancy represents about 90% of all cases (1, 2). Although slow growth rate and excellent outcomes after surgery are usually observed, PTC is prone to spreading to the cervical lymph node (LN), which is closely related to a high locoregional recurrence and distant metastasis (3, 4) and increased risk of mortality (5). As reported in recent studies, the incidence of PTC in cervical LN metastasis was about 30–80%, most commonly to the central neck compartment (5–7).

For patients with a positive LN determined clinically, the therapeutic level VI dissections were recommended by the American Thyroid Association (ATA) (2). However, it remains largely controversial regarding the role of prophylactic central LN dissection (CLND) (8), even though central LN metastasis (CLNM) occurs frequently in PTC. Prophylactic CLND can reduce locoregional recurrence but is still highly risky in the therapy-related morbidity including phrenic nerve palsy, brachial plexus palsy, cranial nerve injury, chyle leak, and parathyroid and laryngeal recurrent nerve injury (8).

Unfortunately, a large proportion of PTC patients were determined clinically negative for nodal involvement in the preoperative evaluation but were detected with CLNM during the operation or pathological analysis (6). According to the ATA, when patients were diagnosed with known or suspected thyroid nodules, cervical LN exploration should be performed before thyroid operation (2). Biopsy is the gold standard to confirm whether an LN is involved. However, considering the intrinsic properties of invasiveness, risk of infection, high cost as well as time-consuming (9), the clinical application of this technique is also limited. Ultrasound (US) examination is a popular technique to assess thyroid nodules and cervical LNs mainly due to its intrinsic properties of real time, convenience, radiation-free, noninvasive, and inexpensive. However, the lack of sufficient image resolution, large dependence on operator's experience, and degree of detail operation may limit its wide applications in the clinic. Moreover, this technique has also been reported to have inaccurate assessment for parapharyngeal and level VII lymph (10). Compared with US, MRI and CT are more objective in the diagnosis of cervical LN metastasis. However, previous studies have investigated the diagnostic accuracy of CT and MRI and failed to prove the robustness of both modalities over US in predicting LN metastasis (11–14). Due to the lack of quantitative analysis, it is not accurate to apply these techniques to predict CLNM. Thus, a noninvasive quantitative method is urgently needed to identify CLNM in PTCs.

Diffusion-weighted imaging (DWI) as a noninvasive functional MRI technique is capable of visualizing the microstructural characteristics of water diffusion in biological tissues (15). The DWI-derived apparent diffusion coefficient (ADC) parameter has shown the potential in differentiating benign from malignant thyroid nodules and predicting tumor stage, tumor grade, tumor aggressiveness, and histological features of thyroid cancer (16–18). However, it remains unknown if DWI approach is also feasible in predicting LN metastasis of PTC.

Thus, the main purpose of the current study was to assess whether DWI with different b values applied, i.e., 300, 500, and 800 s/mm^2^, could preoperatively predict CLNM in PTCs and establish a clinical prediction model.

## Materials and Methods

### Patients

This retrospective study was approved by the Affiliated Hospital of Jiangnan University, and the informed consent for each involved patient was waived. A search in the hospital's database identified 247 patients with surgically confirmed PTCs. Each patient has undergone thyroid DWI–MRI preoperatively at our institute between January 2015 and April 2018. Surgeries of ipsilateral lobectomy + isthmectomy plus ipsilateral CLND were performed for patients with unilateral PTC, and total thyroidectomy plus bilateral CLND were implemented for patients with bilateral PTC. CLND might be prophylactic or therapeutic. A functional lateral LN dissection (LLND) was applied when lateral LN metastasis (LLNM) was found apparent in preoperative MRI measurement or validated with fine-needle aspiration cytology. The exclusion criteria for patients were defined as follows: without CLND, the maximum diameter of the nodal lesion was ≤1 cm (i.e., too small to be identified on MRI), poor-quality DWI with severe susceptibility and patient motion artifacts, and received any other treatment about thyroid prior to MRI examination. Finally, 214 patients with 271 nodules were included in this study.

Clinical and pathological factors were collected retrospectively, namely, gender, age, primary tumor size, multifocality, bilaterality, extrathyroidal extension (ETE), and central LN status. The largest diameter of primary tumor measured by MRI was considered the size of primary tumor. ETE was defined as a visible invasion of surrounding soft tissues by intraoperative findings. With this, all patients were divided into two groups: ETE and no ETE.

### MRI Imaging Techniques

All MRI experiments were implemented at a 1.5-T MRI scanner (GE Signa HD, GE Healthcare Systems, Milwaukee, WI, USA) with an eight-channel high-definition receiver Synergy-Head/Neck phased-array coil. The applied MRI protocols consisted of T1-weighted image (T1WI), T2WI, DWI, and contrast-enhanced MRI. The spin echo-based T1WI sequence [repetition time (TR)/echo time (TE) = 520/14 ms] in axial view and the fast spin echo-based T2WI sequence (TR/TE = 3,500/95 ms) with and without fat suppression in axial and coronal views were employed for imaging acquisitions.

Multiple b values were chosen after assessing the corresponding quality of DWI images and reviewing the literature (18–22). A single-shot spin-echo echo-planar-imaging-based sequence was employed to acquire DWI images in three orthogonal directions. Each of four b values (0, 300, 500, and 800 s/mm^2^) and short T1 inversion recovery (STIR) fat suppression technique were applied. In addition, to ensure the sufficient signal-to-noise ratio (SNR) in DWI, the number of excitations (NEX) = 4 was used. The shimming adjustment has also been properly applied in the thyroid region prior to image acquisition. Therefore, the neck area which is sensitive to local field inhomogeneities can be visualized with accepted image quality.

Of all patients, contrast-enhanced T1WI (TR/TE = 520/14 ms) was obtained with or without fat suppression immediately after the administration of 0.1 mmol/kg gadolinium-diethylenetriamine penta-acetic acid (Gd-DTPA) at an intravenous injection speed of 1.5 ml/s (Magnevist, Schering AG, Germany). The parameters were shown as follows: slice thickness 3 mm, slice gap 1 mm, field of view (FOV) = 40 cm^2^ × 28 cm^2^; matrix size = 256 × 256, NEX = 4. The whole examination was completed within 30 min.

### MRI Data Analysis

All T2W and T1W MRI images were analyzed using ImageJ (National Institutes of Health, Bethesda, MD; http://rsweb.nih.gov/ij/index.html) software. Two well-trained radiologists (YXG and ZQS) respectively with 8 and 10 years of experience in head, neck and DWI assessed all images and reached a consensus. Both reviewers worked independently and were blinded to the clinical information for each patient.

The DWI-derived ADC parametric maps were fitted with a mono-exponential model shown below:

$$\begin{array}{l} \text{S}\left(\text{b}\right)/\text{S}0=\text{exp}\left(-\text{b}\times \text{ADC}\right) \end{array}$$

where S and S0 represent the signal intensities with and without diffusion weighting, respectively, and b was the gradient factor (s/mm^2^).

Overlaying the ADC parametrical maps on contrast-enhanced T1WI, circular or elliptical regions of interest (ROIs) with size of 20–40 mm^2^ were manually chosen in the solid component of the tumor region, avoiding necrotic, hemorrhagic, and cystic areas, with the highest signal intensities in diffusion images at each b value (300, 500, and 800 s/mm^2^). The ADC was calculated at each ROI, and the mean ADC of the three ROIs was used for further analyses.

### Histopathological Analysis

The median time interval between the preoperative MRI examination and radical thyroidectomy or lobectomy was 4 days (range: 1–10 days). Supervised by a senior pathologist with 31 years of experience (CJH), surgical specimens of thyroid tumors taken after surgery were collected. Each surgically resected specimen from tumor was embedded as a tissue block with paraffin and then stained with hematoxylin and eosin (H&E). The H&E section of each thyroid tumor was evaluated by the employed pathologist. Well-established criteria were applied to assess tumor aggressiveness and cervical LN status.

### Statistical Methods

Clinicopathological variables and ADC values respectively obtained by using b values of 300, 500, or 800 s/mm^2^ were analyzed using univariate analysis. Receiver operating characteristic (ROC) curves for the ADC_300_, ADC_500_, and ADC_800_ values were used to differentiate PTCs with from without CLNM. The area under the curve (AUC), sensitivity, specificity, and best cutoff values were determined for each parameter. With the highest AUC, this parameter was considered the best in the differentiation of PTCs with and without CLNM and further involved in multivariate analysis. Subsequently, continuous variables (primary tumor size and the optimal ADC value) were converted into classification variables according to the cutoff values. Binary logistic regression was applied to determine the significance of variables with CLNM. A nomogram was created based on the outcomes of the binary logistic regression to preoperatively assess the probability of CLNM risk. The discriminative ability of the nomogram was quantified by ROC and comparing nomogram-predicted versus observed central LNs of metastasis probability. Bootstraps with 1,000 resamples were used in these analyses.

Interobserver agreement on ADC evaluation was assessed using the intraclass correlation coefficient (ICC) analysis embedded in MedCalc version 12.2.2 (MedCalc Software, Mariakerke, Belgium). The obtained ICC values of 0.00–0.20 were for poor agreement, 0.21–0.40 for fair agreement, 0.41–0.60 for moderate agreement, 0.61–0.80 for strong agreement, and 0.81–1.00 for almost perfect agreement.

For all statistical analyses mentioned above, the significant threshold was set as p = 0.05, and all these analyses were performed with R software (version 3.5.1; http//www.R-projetc.org).

## Results

### Patient Characteristics

Among the 214 patients, 166 females and 48 males were included, with an overall mean age of 47.4 years (range: 24–74 years). The average tumor size was 1.49 ± 0.77 cm, ranging from 1.0 to 4.9 cm. All the PTC patients underwent ipsilateral lobectomy + isthmectomy plus ipsilateral prophylactic CLND (*n* = 168) or total thyroidectomy plus bilateral CLND (*n* = 46). Fifty-four underwent the therapeutic LLND. Based on the surgical pathology and reports, CLNM was found in 100 (46.7%) cases. The demographic and clinicopathological characteristics of the 214 consecutive patients in this study are shown (Table 1).

**Table 1:** Clinicopathological features of patients with PTC according to CLNM.

**Characteristics**	**Without CLNM**	**CLNM**	***P* value**
Gender			0.013
Male	18	30	
Female	96	70	
Age categories			0.191
≥45	76	58	
<45	38	42	
Primary tumor size (cm)	1.30 (1.10–1.50)	1.65 (1.10–2.30)	<0.001
Multifocality			<0.001
No	94	60	
Yes	20	40	
Bilaterality			<0.001
No	100	68	
Yes	14	32	
ETE			<0.001
No	70	22	
Yes	14	62	

*CLNM, central lymph node metastasis; ETE, extrathyroidal extension; PTC, papillary thyroid carcinoma*.

### Quantitative Diffusion-Weighted Imaging Assessment

**Figure 1:**
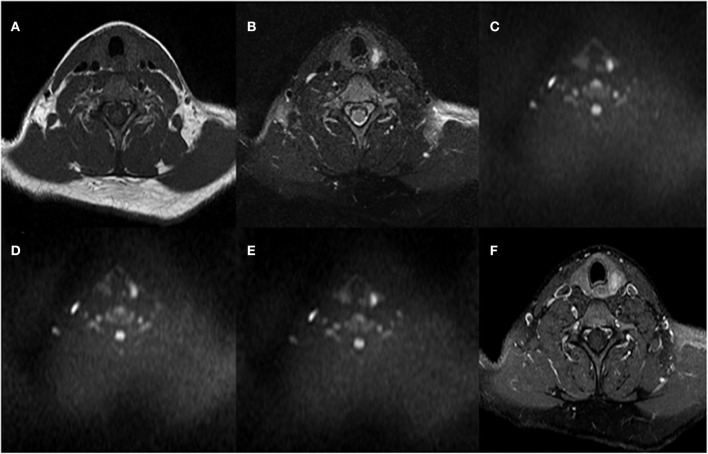
A 32-year-old female with papillary thyroid carcinoma (PTC) in the left thyroid lobe. No central lymph node metastases (CLNMs) were found in histopathologic analysis. **(A)** T1-weighted imaging (T1WI) image, **(B)** Fat-Sat T2WI image, **(C)** the b = 300 s/mm^2^ image, **(D)** 500 s/mm^2^ image, **(E)** 800 s/mm^2^ image, and **(F)** contrast-enhanced T1WI.

**Figure 2:**
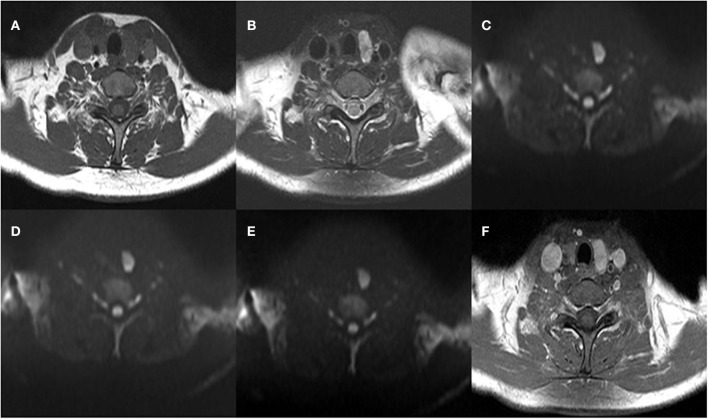
A 39-year-old woman with papillary thyroid carcinoma (PTC) in the left thyroid lobe. Histopathologic analysis revealed central lymph node metastasis (CLNM). **(A)** T1-weighted imaging (T1WI) image, **(B)** Fat-Sat T2WI image, **(C)** the b = 300 s/mm^2^ image, **(D)** 500 s/mm^2^ image, **(E)** 800 s/mm^2^ image, and **(F)** contrast-enhanced T1WI.

The mean ADC values (ADC_300_, ADC_500_, and ADC_800_) of PTCs with and without CLNM are summarized in Table 2. The mean values of ADC_300_, ADC_500_, ADC_800_ were 1.799 ± 0.416 × 10^−3^ mm^2^/s, 1.404 ± 0.307 × 10^−3^ mm^2^/s, and 1.284 ± 0.233 × 10^−3^ mm^2^/s for PTCs with CLNM, respectively. Significantly lower ADC values (ADC_300_, ADC_500_, and ADC_800_) were shown in PTCs with than without CLNM (*p* = 0.015, *p* < 0.001, and *p* < 0.001, respectively). Figures 1, 2 show the ADC maps (ADC_300_, ADC_500_, and ADC_800_) and images of representative PTCs with and without CLNM, respectively.

**Table 2:** Comparison of mean ADC values of CLNM and without CLNM.

**b value (s/mm^**2**^)**	**ADC value (× 10** ^****−3****^ **mm** ^****2****^ **/s, Mean** **±** **SD)**		***P* value**
	**Without CLNM**	**CLNM**	
b = 300	1.927 ± 0.337	1.799 ± 0.416	0.015
b = 500	1.759 ± 0.321	1.404 ± 0.307	<0.001
b = 800	1.439 ± 0.309	1.284 ± 0.233	<0.001

*ADC, apparent diffusion coefficient; CLNM, central lymph node metastasis*.

**Figure 3:**
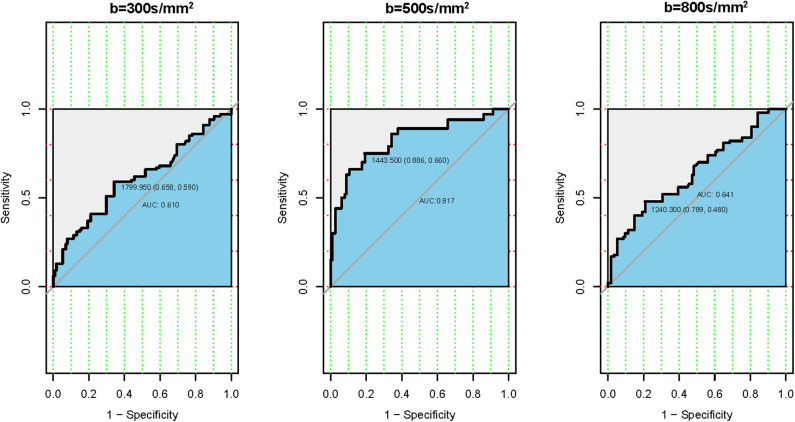
Receiver operating characteristic (ROC) curves according to the mean apparent diffusion coefficient (ADC) values of three different b-values. **(Left)** ROC of b-value 300 s/mm^2^. **(Middle)** ROC of b-value 500 s/mm^2^. **(Right)** ROC of b-value 800 s/mm^2^.

The ROC analysis of the mean ADC_300_, ADC_500_, and ADC_800_ values was also performed for 100 patients with CLNM and 114 patients without CLNM. The resultant AUC of the mean ADC_500_ value (0.817) was higher than both ADC_300_ and ADC_800_ values (0.610, 0.641), indicating a more robust differentiation of CLNM from without CLNM (Figure 3). The corresponding cutoff values of ADC_500_ for discriminating PTCs with and without CLNM was 1.444 × 10^−3^ mm^2^/s, with sensitivity of 88.6% and specificity of 66.0%.

### Establishment and Validation of the Nomogram Model

We analyzed all the variables collected in univariate analysis. In this analysis, all predictors except age showed a significant relationship with CLNM, with *P* < 0.05 (Table 1). Continuous variables (primary tumor size and ADC_500_) were converted into classification variables according to the cutoff values (Table 3). Finally, gender, primary tumor size, multifocality, bilaterality, ETE, and ADC_500_ were entered into the binary logistic regression model. As a result, four predictors were significantly associated with CLNM, namely, gender, primary tumor size, ETE, and ADC_500_ (Table 4).

**Table 3:** Receiver operating characteristic curves of continuous variable.

**Variable**	**Cutoff**	***P* value**	**AUC**
Primary tumor size (cm)	1.65	<0.01	0.716
ADC_300_ (× 10^−3^ mm^2^/s)	1.799	0.015	0.610
ADC_500_ (× 10^−3^ mm^2^/s)	1.444	<0.01	0.817
ADC_800_ (× 10^−3^ mm^2^/s)	1.240	<0.01	0.641

*ADC, apparent diffusion coefficient; AUC, area under the curve*.

**Table 4:** Binary logistic regression model for prediction of CLNM.

**Predictors**	**Odds ratio**	**95% CI**	***P* value**
Gender			
Male	1		
Female	0.13	0.12–0.76	0.011
Primary tumor size (cm)			
<1.65	1		
≥1.65	7.73	3.09–19.29	<0.001
Multifocality			
No	1		
Yes	5.31	0.93–30.27	0.060
Bilaterality			
No	1		
Yes	0.47	0.07–3.16	0.434
ETE			
No	1		
Yes	4.62	2.02–10.60	<0.001
ADC_500_(× 10 ^−3^ mm ^2^ /s)			
<1.444	1		
≥1.444	0.05	0.02–0.12	<0.001

*ADC, apparent diffusion coefficient; CLNM, central lymph node metastasis; ETE, extrathyroidal extension*.

**Figure 4:**
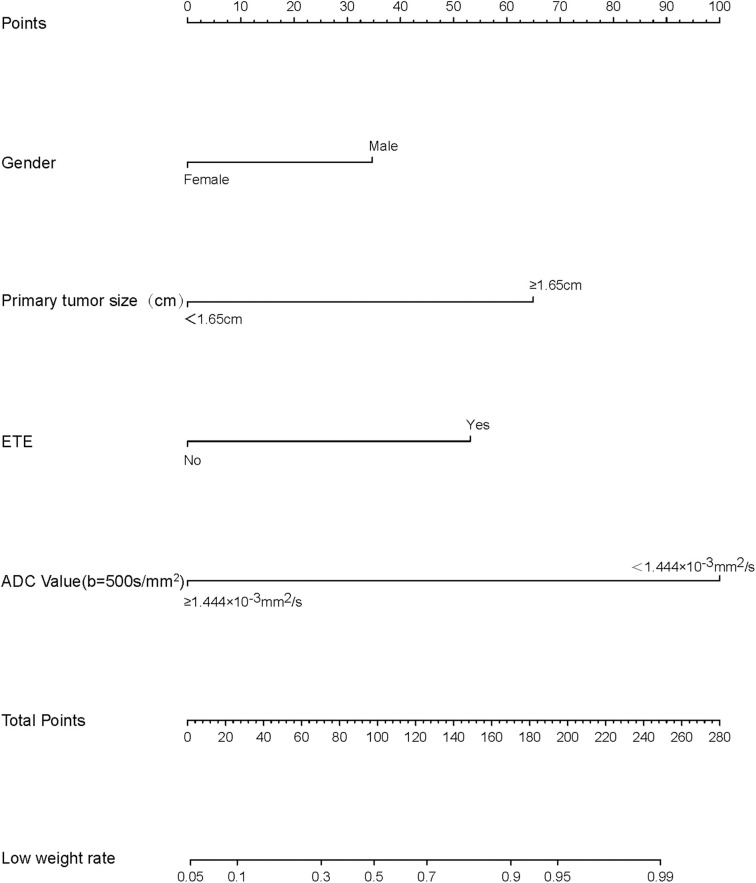
Nomogram of predicting the risk of central lymph node metastasis (CLNM) in papillary thyroid carcinomas (PTCs).

**Figure 5:**
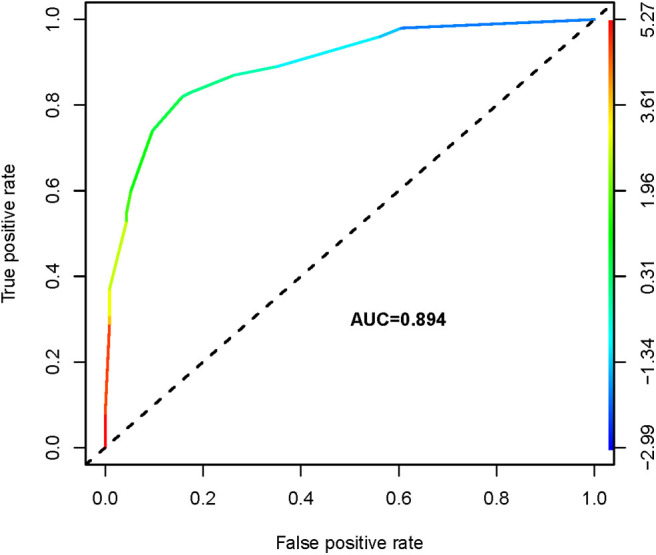
Receiver operating characteristic curve of the nomogram.

**Figure 6:**
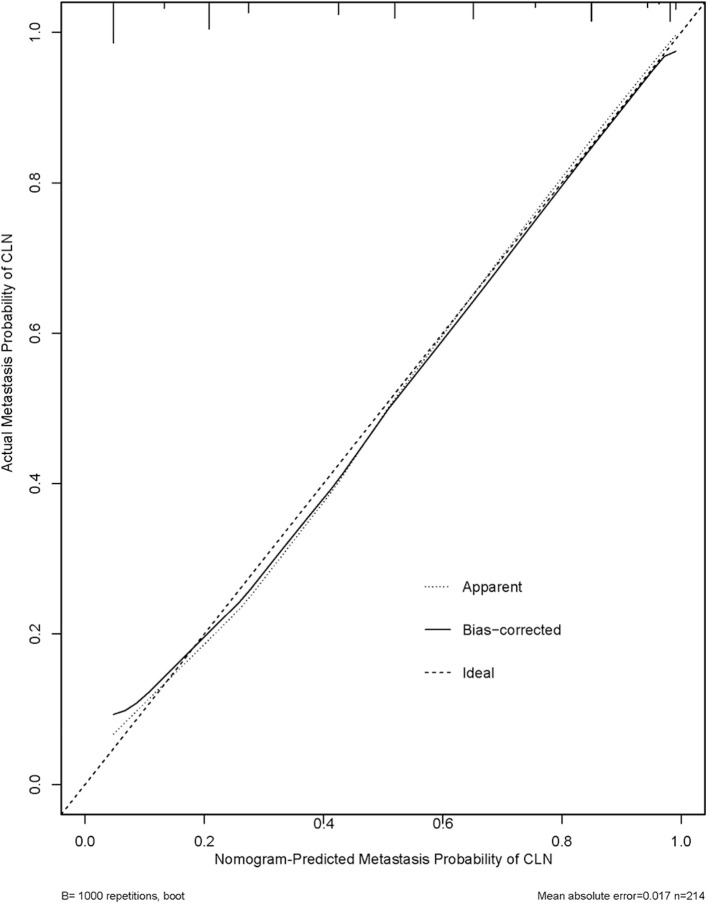
Calibration plots for internal validation of the central lymph node metastasis (CLNM) nomograms. x-axis is nomogram-predicted metastasis probability of lateral neck. y-axis is actual metastasis probability of lateral neck. Dashed line ideal nomogram; dotted line apparent predictive accuracy; solid line calibration estimates from internally validated model.

We thus used these four variables of gender, primary tumor size, ETE, and ADC_500_ as predictors to create a nomogram model (Figure 4). The AUC value of the nomogram is 0.894 (Figure 5). The nomogram was further calibrated using a similar bootstrap resampling procedure (Figure 6). Both predicted and observed metastasis risks of central LN were in good agreement.

### Interobserver Agreement

Strong agreement was separately revealed for the mean ADC_300_, ADC_500_, and ADC_800_ measures with high ICCs of 0.813, 0.859, and 0.842.

## Discussion

As far as we know, no other studies have so far assessed the clinical value of multi-b DWI in predicting CLNM of PTCs. In this study, the derived ADCs using separated b values (ADC_300_, ADC_500_, and ADC_800_) showed significantly lower values in PTCs with than without CLNM. Similar results were also reported in patients with epithelial ovarian cancer (23). The most important findings of the present study were that ADC values (ADC_300_, ADC_500_, and ADC_800_) from DWI–MRI provide a promising quantitative biomarker to evaluate CLNM in PTCs.

As we all know, the LNs of the central neck (level VI) compartment are bordered by the hyoid bone superiorly, suprasternal notch inferiorly, and the carotid sheaths laterally. PTC initially spreads from the thyroid gland to LN of the level VI compartment, which represents the first echelons of lymphatic drainage, followed by the LNs in the lateral neck (levels II, III, and IV) (2). Some recent papers have claimed that for PTCs, poor prognosis relevant factors are extrathyroidal expansion, LN metastasis, and multifocality (3, 24), and CLNM is the most important risk factor for local recurrence (25). Additionally, LN metastasis is strongly correlated with increased cumulative incidence of death caused by thyroid cancer (26, 27). In addition, 28%~33% of the PTCs showed no CLNM in preoperative imaging examination but was confirmed after prophylactic CLND which changed the staging of PTC and the treatment plan after operation (28). Therefore, CLNM of PTCs is a key factor to determine tumor stage, surgical plan, and further treatment after operation. However, accurate preoperative diagnosis of cervical LN metastasis is still difficult.

Conventional imaging techniques with morphological assessment have been so far widely applied in the evaluation of central LN status of PTC preoperatively. US is usually chosen clinically as the first choice for PTC patients to assess cervical LN metastasis, as indicated by the new ATA guidelines (2). A recent meta-analysis revealed that low sensitivity (0.63) was obtained in the preoperative US diagnosis of cervical node metastasis (29). The reason of the low sensitivity may be explained that US depends on operator skill, and the central LNs are located in deep regions and easily affected by thyroid tissue, trachea, and surrounding structures. Several US studies have reported variable and relatively low sensitivity for the assessment of central cervical LN metastasis (12, 13, 30). CT was also not suggested to evaluate PTC mainly because of the uptake of iodine contrast agent and not determined sensitivity in detecting LN metastasis from PTC (13). Compared with US, CT cannot significantly improve the sensitivity of preoperative diagnosis for CLNM (13).

Recent studies indicate that DWI may serve as an effective tool in tumor staging for PTC, as it can provide quantitative and complementary information on the tumor status (17, 18, 31, 32). Conventional MRI is mainly able to provide LN morphology information (33), such as node size, pattern of enhancement, necrosis, signal characteristics, and extranodal extension. Morphological criteria for the evaluation of LNs are simple and easy to be performed, but they are subjective (34). Furthermore, many metastatic cervical LNs of PTC are smaller than 10 mm (35), introducing a difficulty to place a proper ROI on small LN. Schob et al. (32) found that the histogram analysis of DWI is robust in the prediction of lymphatic metastatic spread in thyroid cancer and further explained that LN metastasis is associated with certain characteristics of primary tumor. A previous study reported that the mean ADC values were significantly different between epithelial ovarian cancer with and without LN metastasis (1.01 ± 017 × 10^−3^ mm^2^/s vs. 1.25 ± 0.22 × 10^−3^ mm^2^/s; *p* < 0.001) (23). This result is consistent with our study. The beneficial role of DWI in the differentiation of PTCs with and without CLNM may be linked to PTCs with CLNM showing more diffusion restriction and lower ADC levels than PTCs without CLNM owing to their cellularity. As shown in the current study, the parameter of ADC_500_ showed the best capacity to predict CLNM in PTCs.

In our study, univatiate and multivariate logistic regression analysis were applied to estimate the clinical and pathological variables retrospectively. Univariate analysis showed that CLNM was significantly correlated with gender, primary tumor size, multifocality, bilateral and ETE. Similar results were obtained in a recent study (25). We included the ADC_500_ value and univariate regression analysis into multivariate regression analysis. The results showed that male gender, primary tumor size, ETE, and ADC_500_ value were independent risk factors for predicting LN metastasis in the central region. Also, for the first time, we used a nomogram model with DWI-derived parameter ADC and clinicopathological features to predict the CLNM of PTCs prior to surgery. In total, four predictors, i.e., gender, primary tumor size, ETE, and ADC_500_ value, were included in the nomogram. The sensitivity of 83.0% and specificity of 87.7% were thus obtained. Using a binary logistic regression model, the accuracy of the nomogram was 85.5%. We hope to establish an intuitive, practical, and credible clinical prediction model to preoperatively assess the risk of CLNM in PTCs and help radiologists predict the risk of CLNM in PTCs before surgical operation.

Our study has several limitations. First, the microcystic structure may have affected our results even though the cystic portions of PTCs have been carefully avoided in ROI prescription. Secondly, each ROI was manually drawn within the solid component area of the tumors on ADC maps. These ROIs may not reflect the overall tumor characteristics. Thirdly, our study was limited by its retrospective nature. A further multicenter prospective study with a larger cohort is requested.

In summary, this study demonstrated that DWI can provide a noninvasive valuable information to predict CLNM of PTCs before surgery. The clinical predictive model of the nomogram is able to provide an accurate preoperative evaluation of CLNM risk in PTC patients. This may help clinicians in the clinical diagnosis of patients with CLNM. Further research with larger numbers of patients is required to confirm our results.

## Data Availability Statement

The datasets used and/or analyzed during the current study are available from the corresponding author on reasonable request.

## Author Contributions

HZ, SH, XW, WL, JH, ZS, and YG contributed conception and design of the study. SH and HZ supervised the project. HZ, XW, JH, and WL organized the database. HZ, XW, ZS, and YG acquired, analyzed, and interpreted the patient date. HZ and SH wrote the first draft of the manuscript. WD gave technical support and conceptual advice. All authors contributed to manuscript revision, read, and approved the submitted version.

## Conflict of Interest

WD was employed by the company GE Healthcare China. The remaining authors declare that the research was conducted in the absence of any commercial or financial relationships that could be construed as a potential conflict of interest.
